# Comparative outcomes of transcatheter aortic valve replacement in bicuspid vs. tricuspid aortic valve stenosis patients: insights from the SWEDEHEART registry

**DOI:** 10.1016/j.ijcha.2025.101705

**Published:** 2025-05-14

**Authors:** Antros Louca, Petur Petursson, Joakim Sundström, Araz Rawshani, Henrik Hagström, Magnus Settergren, Stefan James, Sasha Koul, Kristofer Skoglund, Dan Ioanes, Sebastian Völz, Anna Myredal, Oskar Angerås, Truls Råmunddal

**Affiliations:** a Department of Cardiology Sahlgrenska University Hospital, Gothenburg, Sweden; b Department of Molecular and Clinical Medicine, Institute of Medicine, University of Gothenburg, Gothenburg, Sweden; c Heart Centre, Umeå University Hospital, Umeå, Sweden; d Department of Public Health and Clinical Medicine, Umeå University, Umeå, Sweden; e Department of Cardiology, Karolinska University Hospital and Karolinska Institutet, Sweden; f Department of Medical Sciences, Cardiology, Uppsala University, Sweden; g Department of Cardiology, Lund University, Skåne University Hospital, Lund, Sweden

**Keywords:** Transcatheter Aortic Valve Replacement, Bicuspid Aortic Valve, Aortic Valve Stenosis

## Abstract

**Background:**

Limited data exist on transcatheter aortic valve replacement (TAVR) outcomes in patients with bicuspid aortic valve (BAV) stenosis. This study compared TAVR outcomes in BAV versus tricuspid aortic stenosis.

**Methods:**

This observational study included all patients who underwent TAVR in Sweden from 2016 to 2022, excluding those with pure aortic insufficiency and valve-in-valve procedures. Only Evolut-, SAPIEN-, ACURATE-, and Portico/Navitor-family devices were included. A doubly robust method was used, combining propensity score estimation and multivariable regression.

**Results:**

Among 7,095 patients, 577 (8.1 %) had BAV stenosis. The mean EUROSCORE II-predicted mortality risk was 3.8 % for BAV and 4.5 % for TAV. BAV patients were younger, predominantly male, and had fewer comorbidities but higher baseline aortic valve gradients, larger annulus diameters, and more reduced ejection fraction.

After matching, 30-day mortality and all-cause mortality (median follow-up: 690 days) were similar between BAV and TAV patients (p = 0.8 for both). While BAVs had numerically lower technical success per VARC-3 criteria, this was not statistically significant (p = 0.08). However, BAV patients had lower device success (aOR = 0.8, p = 0.04) and a higher incidence of post-TAVR pacemaker implantation (aOR = 1.76, 95 % CI: 1.14–2.58, p = 0.007). No significant differences were observed in prosthesis-patient mismatch (p = 0.3), paravalvular leakage (p = 0.6), stroke (p = 0.3), or post-TAVR gradients (p > 0.9).

**Conclusion:**

TAVR in BAV patients yields similar mortality and hemodynamic outcomes as in TAV patients. However, BAVs are associated with lower device success and higher pacemaker rates. While TAVR is a viable alternative to SAVR, treatment should be individualized, especially in younger BAV patients, considering lifetime management and coronary access.

## Graphical abstract

**Figure:**
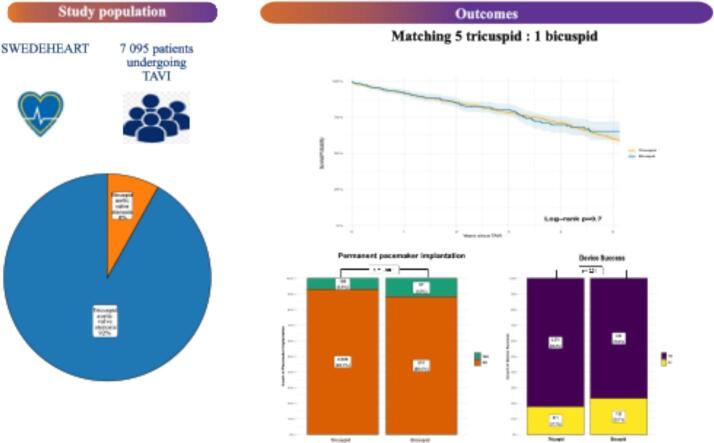

## 1 Introduction

Transcatheter aortic valve replacement (TAVR) today constitutes a cornerstone therapy for patients with aortic stenosis with ever increasing world-wide volumes [1]. Initially, TAVR was approved only for patients who were inoperable or at high risk for surgery [2]. Over the years, as studies demonstrated that TAVR outcomes were comparable or even superior to surgical aortic valve replacement (SAVR) for elderly patients with tricuspid AV (TAV) stenosis [[3], [4], [5], [6]], guidelines have expanded. These now include recommendations for TAVR in surgically intermediate-risk patients and in older low surgical-risk patients [7,8].

Bicuspid aortic valve (BAV) is the most common congenital valvular heart disease with an estimated prevalence of 0.5 % to 1.4 % [9,10] and is associated with early degeneration leading to aortic stenosis or regurgitation [11]. These patients, often younger, have been excluded from most of the trials assessing TAVR outcomes due to their usually low risk and age [5,6]. The only randomized study including BAV patients, NOTION 2 [12], reported higher rates of death and stroke in the BAV group. However, this study was underpowered, and its findings should be interpreted with caution. Therefore, results of TAVR in patients with TAV cannot be extrapolated to patients with BAV. The use of TAVR in BAV patients is additionally on the rise as both the Evolut and Sapien valves have gained the European Conformity (CE) mark and the prior restrictions on their use in BAV in the USA have been lifted [13,14]. In addition, as TAVR indications expand to include low-risk patients, more individuals with BAV will become eligible to be treated with TAVR.

The objective of this study was to evaluate the outcomes of TAVR in patients with aortic stenosis with BAV morphology and compare them to patients with TAV morphology using data from a nationwide registry.

## 2 Methods

### 2.1 Data sources and patient selection

The study population for this observational study was derived from the SWENTRY registry (SWEdish traNscatheter cardiac intervention regisTRY). The SWENTRY is part of the SWEDEHEART (Swedish Web-system for Enhancement and Development of Evidence-based Care in Heart Disease Evaluated According to Recommended Therapies) [15] registry, which provides complete nationwide coverage of the Swedish population.

**Fig. 1:**
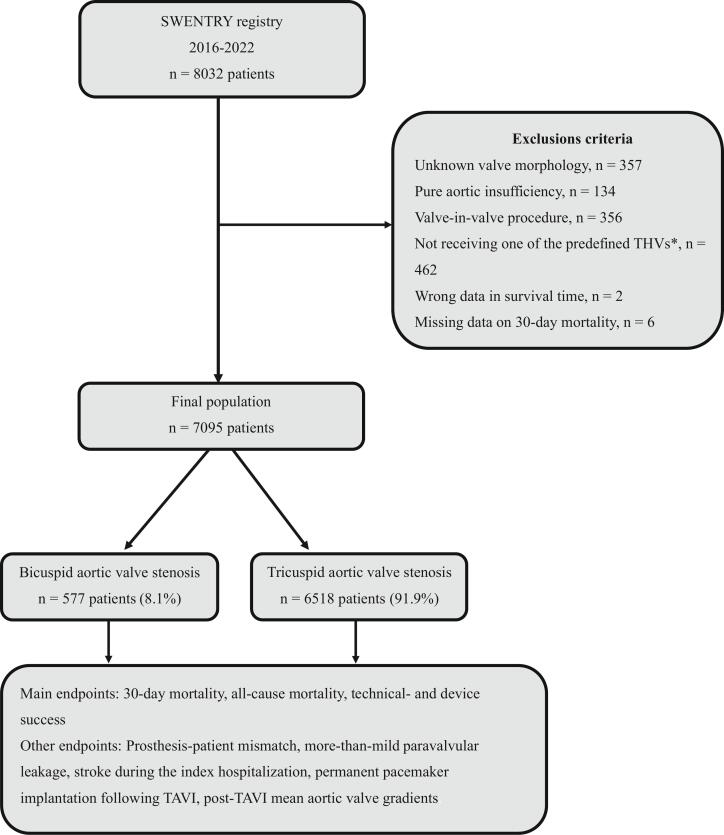
Patient selection process. *Only valves from the Evolut family, Sapien family, Portico/Navitor family, and Acurate family were included.

This study included all patients who underwent TAVR in Sweden between January 1, 2016, and September 30, 2022. Only patients receiving one of the major transcatheter heart valves from the second-generation onward − including the Evolut family, Sapien family, Portico/Navitor family, or Acurate family − were included. Exclusion criteria included patients undergoing TAVR for pure aortic insufficiency, those undergoing valve-in-valve procedures (previous TAVR or SAVR), and cases with missing 30-day mortality data (Fig. 1).

### 2.2 Outcomes

The main outcomes included mortality endpoints, specifically all-cause mortality for the median follow-up time of 690 days and 30-day mortality due to any cause, as well as device success and technical success during the index hospitalization, as defined by the VARC-3 criteria [16] (Supplementary Table 1 for definitions).

Other outcomes included the incidence of prosthesis-patient mismatch (PPM), more-than-mild paravalvular leak (PVL), stroke during the index hospitalization, the need for permanent pacemaker implantation (PPI) following TAVR, and post-TAVR mean aortic valve gradients.

### 2.3 Statistical analysis

Continuous variables are presented as mean ± standard deviation. The Student’s *t*-test or Welch *t*-test was used to compare continuous variables between two groups, depending on the variables’ variance as determined by Levene’s test. Categorical variables are expressed as counts and percentages, with group differences assessed using the χ^2^ test or Fisher’s exact test.

Missing data in the covariates (Supplementary Fig. 1) were assessed to be missing at random (MAR) and were imputed using a random forest algorithm [17], with reduced weighting assigned to observations with a high proportion of missing values to limit their influence during the imputation process.

A double robust approach was employed to compare the outcomes in the BAV and TAV groups. First, a propensity score was calculated using the Covariate Balancing Propensity Score (CBPS) algorithm [18] based on the following covariates: baseline characteristics (year of TAVR, age at TAVR, sex, body mass index [BMI]), comorbidities (hypertension, diabetes mellitus, chronic kidney disease [eGFR ≤ 60 mL/min/1.73 m^2^], atrial fibrillation, chronic pulmonary disease, peripheral vascular disease), medical history (previous myocardial infarction, previous PCI, previous CABG, previous cerebrovascular incident, prior pacemaker implantation), valve characteristics and echocardiographic features (aortic valve area, mean aortic valve gradient, maximum aortic valve gradient, aortic annulus diameter, systolic pulmonary artery pressure, ejection fraction, moderate/severe mitral insufficiency, moderate/severe aortic insufficiency), laboratory values and clinical features (NT-proBNP, NYHA functional class III or IV, porcelain aorta, thorax deformity, unfavourable anatomy) as well as procedural characteristics (access site, urgency of TAVR procedure, BEV or SEV).

A 5:1 (5 TAV:1 BAV) greedy matching [19] (nearest neighbor matching) with no replacement was used for matching. Covariate balance was assessed using standardized mean differences (SMD) and the Kolmogorov-Smirnov statistic, with excellent balance defined as SMDs below 0.10 and Kolmogorov-Smirnov statistics below 0.05. Variance ratios were also evaluated for continuous variables, with values below 2 indicating acceptable balance. Subsequently, multivariable regression was applied to the matched population using covariates not perfectly balanced (Supplementary Table 3) to account for any residual confounding. Marginal odds ratios and confidence intervals were calculated through G-computation using cluster-robust standard errors. Kaplan-Meier survival curves were constructed on the propensity score matched population, with p-values obtained through the log-rank test. Cox regression models were used for all-cause mortality, and the proportional hazard assumption was tested via Schoenfeld residuals.

Sensitivity analyses were performed to address missing observations in the outcomes (Supplementary Table 2). Outcomes were first analysed using a complete case approach. Subsequently, missing outcomes were imputed using multiple imputations via chained equations, employing predictive mean matching. Each imputed dataset was then re-analysed separately. Finally, the results from these multiple analyses were combined using Rubin’s Rules [20].

To ensure the robustness of the findings, a sensitivity analysis was conducted using Inverse Probability of Treatment Weighting (IPTW). Weights were calculated for the BAV and TAV groups based on the above-mentioned covariates, employing CBPS [18]. These weights were incorporated into a multivariable regression model to minimize residual confounding [21].

All p-values were two-sided, with values below 0.05 considered statistically significant. Analyses were conducted in R (R Foundation for Statistical Computing, version 4.3.1).

This study was conducted in accordance with the principles of the Declaration of Helsinki, and it was approved by the Swedish Ethical Review authority.

## 3 Results

**Fig. 2:**
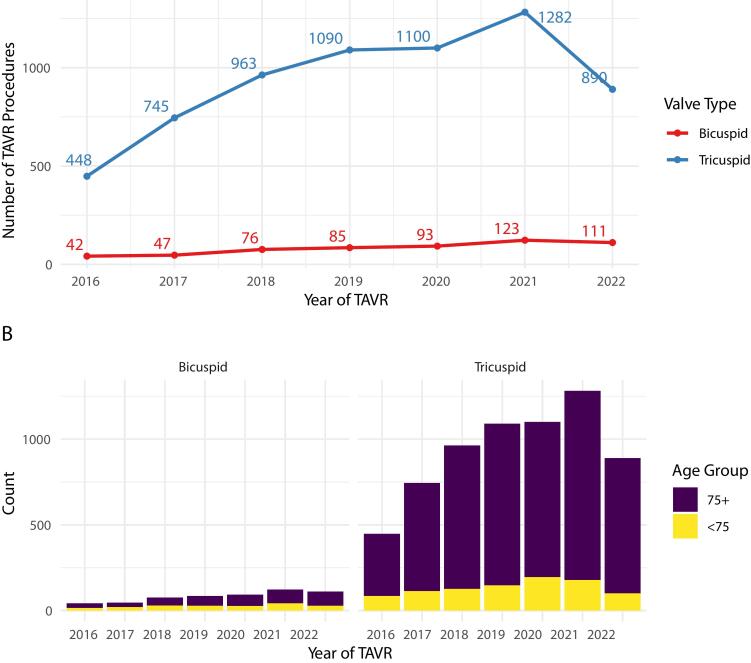
Annual TAVR procedures by valve type (Panel A) and stratified by age (Panel B). Note: Data include only TAVR procedures conducted up to September 2022.

Over the years, there has been a rising trend in TAVR procedures among both BAV and TAV stenosis groups in Sweden (Fig. 2, Panel A). While TAVR has been predominantly utilized in patients over 75, there is an emerging trend of increasing use among those under 75 years of age for both BAV and TAV morphology (Fig. 2, Panel B).

### 3.1 Baseline characteristics

The study included 7,095 patients, of whom 577 (8.1 %) underwent TAVR for BAV stenosis. The mean [SD] EUROSCORE II-predicted risk of mortality was 3.1 3.8 % [4.2 %] for BAV and 4.5 % [4.5 %] for TAV. Those with BAV were younger (76.8 ± 7.8 years vs. 80.9 ± 6.4 years, p < 0.001) and had a higher proportion of males (60 % vs. 53 %, p = 0.004) compared to patients with TAV stenosis. BAV patients also had a lower prevalence of comorbidities such as hypertension (69 % vs. 78 %, p < 0.001), diabetes mellitus (21 % vs. 26 %, p = 0.008), chronic kidney disease (42 % vs. 50 %, p < 0.001), atrial fibrillation (29 % vs. 37 %, p < 0.001) and a prior history of PCI (19 % vs 28 %, p < 0.001). No significant differences were observed in the incidence of peripheral vascular disease (p = 0.08), chronic pulmonary disease (p = 0.5), prior cerebrovascular incident (p = 0.13) or previous pacemaker (p = 0.8).

Patients with BAVs demonstrated higher baseline mean aortic valve gradients (49.3 ± 14.1 mmHg vs. 47.2 ± 13.6 mmHg, p < 0.001), a higher prevalence of reduced ejection fraction as defined by EF under 40 % (20 % vs. 14 %, p = 0.004), and a larger aortic annulus diameter (25.7 ± 2.6 mm vs. 24.7 ± 2.3 mm, p < 0.001). BAV patients also exhibited lower systolic pulmonary artery pressures (26.8 ± 21.3 mmHg vs. 31.2 ± 21.2 mmHg, p < 0.001). Additionally, these patients had a lower prevalence of porcelain aorta (2.3 % vs. 4.6 %, p = 0.008) but a higher incidence of thoracic deformity (3.3 % vs. 2.0 %, p = 0.04). (Table 1).

**Table 1:** Baseline characteristics of the TAVR population.

Characteristic	Overall N = 7,095^a^	Bicuspid aortic stenosis N = 577^a^	Tricuspid aortic stenosis N = 6,518^a^	p-value^b^	Missing
Age at TAVI	80.6 (6.6)	76.8 (7.8)	80.9 (6.4)	**<0.001**	0
Sex				**0.004**	0
Male	3,819 (54 %)	344 (60 %)	3,475 (53 %)		
Female	3,276 (46 %)	233 (40 %)	3,043 (47 %)		
BMI (kg/m^b^)	26.8 (5.0)	26.4 (5.3)	26.9 (5.0)	**0.04**	44
NYHA functional status III or IV	5,459 (77 %)	417 (72 %)	5,042 (77 %)	**0.005**	8
EUROSCORE-II predicted mortality (%)	4.4 (4.4)	3.8 (4.2)	4.5 (4.5)	**< 0.001**	0

**Comorbidities**					
Hypertension	5,498 (77 %)	400 (69 %)	5,098 (78 %)	**<0.001**	0
Diabetes Mellitus	1,840 (26 %)	123 (21 %)	1,717 (26 %)	**0.008**	1
CKD (eGFR < 60 mL/min/1.73 m2)	3,505 (50 %)	242 (42 %)	3,263 (50 %)	**<0.001**	20
Peripheral vascular disease	1,105 (16 %)	75 (13 %)	1,030 (16 %)	0.08	0
Chronic pulmonary disease	1,172 (17 %)	89 (15 %)	1,083 (17 %)	0.5	0
Atrial fibrillation	2,579 (36 %)	168 (29 %)	2,411 (37 %)	**<0.001**	0

**Past history**					
Myocardial infarction within 3 months	245 (3.5 %)	18 (3.1 %)	227 (3.5 %)	0.6	0
History of PCI	1,906 (27 %)	111 (19 %)	1,795 (28 %)	**<0.001**	0
Prior cardiac surgery+	812 (11 %)	45 (8 %)	767 (12 %)	**0.03**	0
Prior cerebrovascular incident	785 (11 %)	53 (9.2 %)	732 (11 %)	0.13	0
Previous pacemaker	733 (10 %)	58 (10 %)	675 (10 %)	0.8	2

**Valve characteristics**					
Aortic valve area (cm^b^)	0.7 (0.2)	0.7 (0.2)	0.7 (0.2)	0.8	1,630
Mean aortic valve gradient (mmHg)	47.4 (13.6)	49.3 (14.1)	47.2 (13.6)	**<0.001**	42
Maximum aortic valve gradient (mmHg)	77.1 (21.0)	80.7 (22.8)	76.8 (20.8)	**<0.001**	810
Left ventricular ejection fraction				**0.004**	429
≥50 %	4,671 (70 %)	365 (67 %)	4,306 (70 %)		
41–49 %	1,011 (15 %)	75 (14 %)	936 (15 %)		
≤40 %	984 (15 %)	107 (20 %)	877 (14 %)		
Moderate/severe aortic valve insufficiency	575 (8.3 %)	53 (9.4 %)	522 (8.2 %)	0.3	164
Moderate/severe mitral valve insufficiency	790 (11 %)	49 (8.8 %)	741 (12 %)	**0.04**	195
Annulus diameter (mm)	24.6 (2.4)	25.7 (2.6)	24.5 (2.3)	**<0.001**	9
Pulmonary hypertension	30.9 (21.3)	26.8 (21.3)	31.2 (21.2)	**<0.001**	0

**Anatomical features**					
Porcelain aorta	315 (4.4 %)	13 (2.3 %)	302 (4.6 %)	**0.008**	8
Thorax deformity	150 (2.1 %)	19 (3.3 %)	131 (2.0 %)	**0.04**	2
Unfavorable anatomy after CABG *	367 (5.2 %)	22 (3.8 %)	345 (5.3 %)	0.1	2

BMI: body mass index, CKD: Chronic Kidney Disease, eGFR = estimated Glomerular Filtration Rate, HFmrEF: Heart Failure with mildly reduced Ejection Fraction, HFrEF: Heart Failure with reduced Ejection Fraction, NYHA: New York Heart Association, PCI: Percutaneous Coronary Intervention.

a Mean (SD); n (%).

b Two Sample *t*-test; Pearson’s Chi-squared test; Welch Two Sample *t*-test.

+ History of CABG, aortic surgery, valve surgery (excluding aortic valve procedures), heart transplantation, or arrhythmia surgery.

* Risk for injury of patent bypass grafts.

### 3.2 Matching

**Fig. 3:**
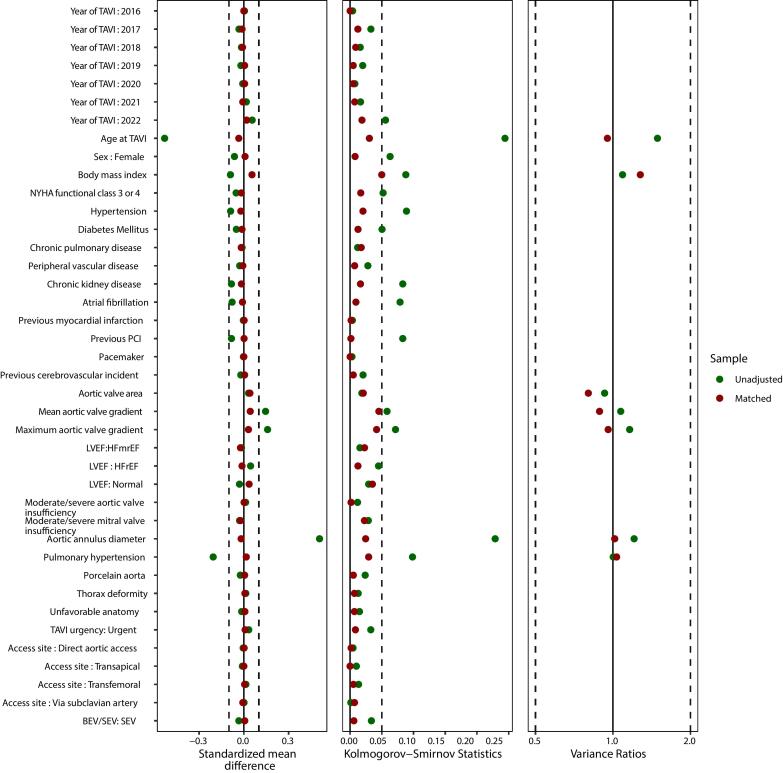
Comparison of covariate balance in the selected population before and after matching.

Matching between BAV and TAV achieved good balance across all covariates, with standardized mean differences (SMDs) below 0.10 for all variables and variance ratios below 2 for all continuous variables. The Kolmogorov-Smirnov statistic was below 0.05 for all variables except for BMI (0.06) (Fig. 3 and Supplementary Table 3).

### 3.3 Procedural characteristics

No significant differences were observed in the access site, with the transfemoral approach being the predominant method for both BAV (95 %) and TAV (94 %) patients. However, a higher proportion of BAV patients underwent non-elective TAVR (15 % vs. 12 %, p = 0.02). BAV patients were more likely to undergo predilatation (69 % vs. 59 %, p < 0.001) and post-dilatation (31 % vs. 24 %, p < 0.001). Balloon-expandable (BEV) and self-expanding valves (SEV) were implanted at similar rates (p = 0.1), although BAV patients generally required larger prosthesis sizes. Additionally, BAV patients received higher amounts of contrast during TAVR (67.75 ± 48.99 mL vs. 59.28 ± 41.43 mL, p < 0.001) and had longer radiation times (19.30 ± 12.24 min vs. 16.45 ± 10.09 min, p < 0.001) (Table 2).

**Table 2:** Procedural characteristics of the TAVR population.

Characteristic	Bicuspid aortic stenosis N = 577^a^	Tricuspid aortic stenosis N = 6,518^a^	p-value^b^	Missing
Access site			0.4	0
Transfemoral	551 (95 %)	6,137 (94 %)		
Via subclavian artery	14 (2.4 %)	150 (2.3 %)		
Transapical	8 (1.4 %)	155 (2.4 %)		
Direct aortic access	4 (0.7 %)	76 (1.2 %)		
TAVR urgency			**0.02**	6
Elective	488 (85 %)	5,744 (88 %)		
Urgent	87 (15 %)	770 (12 %)		
Predilatation	397 (69 %)	3,825 (59 %)	**<0.001**	0
Post-dilatation	179 (31 %)	1,540 (24 %)	**<0.001**	6
BEV/SEV			0.1	0
SEV	303 (53 %)	3,642 (56 %)		
BEV	274 (47 %)	2,876 (44 %)		
Transcatheter heart valve			**<0.001**	0
Sapien	274 (47 %)	2,876 (44 %)		
Evolut	188 (33 %)	1,717 (26 %)		
Acurate	107 (19 %)	1,618 (25 %)		
Portico/Navitor	8 (1.4 %)	307 (4.7 %)		
Prosthesis size (mm)			**<0.001**	3
20	3 (0.5 %)	41 (0.6 %)		
23	55 (9.5 %)	1,235 (19 %)		
25	40 (6.9 %)	633 (9.7 %)		
26	115 (20 %)	1,550 (24 %)		
27	61 (11 %)	850 (13 %)		
29	214 (37 %)	1,732 (27 %)		
34	89 (15 %)	474 (7.3 %)		
Oversizing by ≥ 15 %*	150 (26 %)	1,674 (26 %)	0.9	12
Contrast amount (ml)	67.75 (48.99)	59.28 (41.43)	**<0.001**	12
Radiation dose (Gy)	2,770.47 (6,723.40)	2,519.44 (4,910.44)	0.4	276
Radiation time (minutes)	19.30 (12.24)	16.45 (10.09)	**<0.001**	17

*Theoretical device oversizing was defined using the following calculation: ([valve diameter − annulus diameter] × 100)/annulus diameter.

BEV: ballon expandable valve, SEV: self-expandable valve.

a n (%); Mean (SD).

b Fisher’s exact test; Two Sample *t*-test; Welch Two Sample *t*-test.

### 3.4 Outcomes

**Fig. 4:**
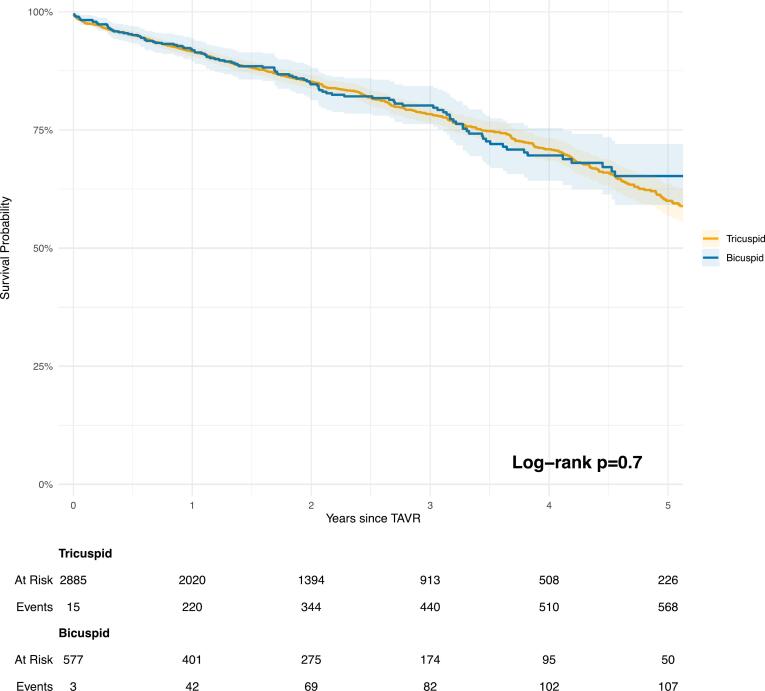
Kaplan-Meier curves for all-cause mortality in the matched population.

The median follow-up time in the unmatched population was 675 days for BAV patients (IQR 289–1208 days) and 779 days (IQR 351–1318 days) for TAV patients (p = 0.04). Regarding mortality endpoints, no statistically significant differences were observed between BAV and TAV patients for 30-day mortality due to any cause (adjusted odds ratio [aOR]: 0.90, 95 % CI: 0.37–2.22, p = 0.8) or all-cause mortality (hazard ratio [HR]: 1.03, 95 % CI: 0.80–1.33, p = 0.8) (Table 3). Kaplan-Meier curves up to 5 years (Fig. 4) also revealed no significant differences (log-rank test p = 0.7). A trend towards reduced technical success was noted in BAV compared to TAV, but this did not reach statistical significance (aOR = 0.7, 95 % CI 0.49–1.04, p = 0.08) (Table 3 and Supplementary Table 4 for comparison of individual technical success endpoints). Although exploratory in nature, BAV patients demonstrated a numerically higher incidence of vascular complications and major bleeding events compared to those with TAV. Additionally, a greater proportion of BAV patients required an additional valve during the procedure, and valve embolization occurred more frequently in the BAV group. BAV was associated with reduced device success during index hospitalization compared to TAV with an aOR of 0.8 (95 % CI: 0.57–0.98, p = 0.04) (Table 3 and Supplementary Table 5 for comparison of device success individual endpoints).

**Table 3:** Comparison of outcomes between unadjusted and propensity score-matched cohorts for bicuspid versus tricuspid aortic stenosis.

Outcome	Unadjusted cohort		Propensity score-matched cohort			
	Tricuspid aortic stenosis N = 6,518	Bicuspid aortic stenosis N = 577	Tricuspid aortic stenosis N = 2,885	Bicuspid aortic stenosis N = 577	Marginal OR/HR/Estimate* (95 % CI)	p-value
30 days mortality	122 (1.9 %)	10 (1.7 %)	58 (2.0 %)	10 (1.7 %)	0.90(0.37, 2.22)	0.8
All-cause mortality	1,502 (23 %)	113 (20 %)	595 (21 %)	113 (20 %)	1.03(0.80, 1.33)	0.8
Technical success	5,996 (92 %)	511 (89 %)	2,643 (92 %)	511 (89 %)	0.7(0.49, 1.04)	0.08
Device success	5,351 (82 %)	444 (77 %)	2,374 (82 %)	444 (77 %)	0.8(0.57, 0.98)	**0.04**
Prosthesis-patient mismatch	162 (2.5 %)	13 (2.3 %)	73 (2.5 %)	13 (2.3 %)	0.69(0.32, 1.49)	0.3
More than mild PVL	162 (2.5 %)	25 (4.3 %)	78 (2.7 %)	25 (4.3 %)	1.2(0.64, 2.26)	0.6
Stroke during index hospitalization	60 (0.9 %)	3 (0.5 %)	22 (0.8 %)	3 (0.5 %)	0.5(0.12, 2.01)	0.3
Pacemaker following TAVR (complete case analysis)	462 (7.1 %)	67 (12 %)	188 (6.9 %)	67 (12 %)	1.76(1.17, 2.66)	**0.007**
Pacemaker following TAVR (multiple imputation datasets)	462 (7.1 %)	67 (12 %)	−	−	1.62(1.27, 1.98)	**<0.001**
Post-TAVR mean average gradient (complete case analysis)	9.0 (7.0, 13.0)	10.0 (7.0, 13.0)	9.5 (7.0, 13.0)	10.0 (7.0, 13.0)	0.01 (−1.06, 1.03)	> 0.9
Post-TAVR mean average gradient (multiple imputation datasets)	9.0 (7.0, 13.0)	10.0 (7.0, 13.0)	−	−	0.44 (−0.32, 1.20)	0.2

Results reported as number of events (%), OR; HR or estimates (95% CI) and p-values.

*Tricuspid aortic stenosis serves as the reference.

The values in bold represent differences between groups with p < 0.05.

CI: confidence interval; HR: Hazard ratio; OR: odds ratio;

Hemodynamically, no significant differences were observed between the two valve anatomies regarding post-TAVR mean average gradients (9.5 mmHg for TAV vs 10.0 mmHg for BAV, p-value > 0.9 for complete case analysis, p-value = 0.2 for multiple imputation), the incidence of PPM (2.5 % for TAV vs 2.3 % for BAV, p-value = 0.3), or more than mild PVL (2.7 % for TAV vs 4.3 % for BAV, p-value = 0.6). Additionally, the occurrence of stroke during hospitalization did not differ significantly between the two groups (0.8 % for TAV vs 0.5 % for BAV, p-value = 0.3). However, BAV anatomy had a higher incidence of PPI following TAVR (aOR 1.76, 95 % CI: 1.17, 2.66, p = 0.007 for complete case analysis and aOR 1.62, 95 % CI: 1.27 – 1.98, p < 0.001 for the imputed datasets) (Table 3).

### 3.5 Sensitivity analysis

Sensitivity analysis using IPTW demonstrated no statistically significant difference in mortality between BAV and TAV, with no differences observed in 30-day mortality (p = 0.9) or all-cause mortality (p > 0.9). Additionally, no significant differences were found regarding PPM (p > 0.9), moderate or severe PVL (p = 0.09), stroke during hospitalization (p = 0.3), or post-TAVR mean pressure gradients (p = 0.2).

However, technical success was significantly lower in the BAV group (aOR 0.7, 95 % CI: 0.49–0.86, p = 0.002), as was device success (aOR 0.7, 95 % CI: 0.56–0.86, p = 0.001). Furthermore, sensitivity analysis indicated a higher likelihood of permanent pacemaker implantation in BAV patients compared to TAV patients (aOR 1.66, 95 % CI: 1.33–1.98, p < 0.001) (Table 4).

**Table 4:** Outcomes in the unadjusted population and after adjustment with inverse probability of treatment weighting (IPTW).

Outcome	Unadjusted cohort		IPTW- adjusted			
	Tricuspid aortic stenosis N = 6,518	Bicuspid aortic stenosis N = 577			Marginal OR/HR/Estimate* (95 % CI)	p-value
30 days mortality	122 (1.9 %)	10 (1.7 %)			0.90(0.48, 1.83)	0.9
All-cause mortality	1,502 (23 %)	113 (20 %)			1.01(0.82, 1.24)	>0.9
Technical success	5,996 (92 %)	511 (89 %)			0.7(0.49, 0.86)	**0.002**
Device success	5,351 (82 %)	444 (77 %)			0.7(0.56, 0.86)	**0.001**
Prosthesis-patient mismatch	162 (2.5 %)	13 (2.3 %)			0.98(0.56, 1.72)	>0.9
More than mild PVL	162 (2.5 %)	25 (4.3 %)			1.5(0.94, 2.41)	0.09
Stroke during index hospitalization	60 (0.9 %)	3 (0.5 %)			0.5(0.12, 2.01)	0.3
Pacemaker following TAVR (multiple imputation datasets)	462 (7.1 %)	67 (12 %)			1.66(1.33, 1.98)	**<0.001**
Post-TAVR mean average gradient (multiple imputation datasets)	9.0 (7.0, 13.0)	10.0 (7.0, 13.0)			0.4 (−0.23, 1.03)	0.2

Results reported as number of events (%), OR; HR or estimates (95% CI) and p-values.

*Tricuspid aortic stenosis serves as the reference.

The values in bold represent differences between groups with p < 0.05.

CI: confidence interval; HR: Hazard ratio; OR: odds ratio;

## 4 Discussion

In this observational, registry-based study of propensity-matched patients with aortic stenosis who underwent TAVR, there was no significant difference in 30-day mortality due to any cause, all-cause mortality or stroke risk between patients with BAV and those with TAV. However, BAV patients exhibited a lower likelihood of device success and a trend toward reduced technical success, with sensitivity analysis using IPTW indicating a significant reduction in technical success among this group. Additionally, BAV patients faced a higher risk of requiring PPI. Hemodynamically, both valve morphologies showed comparable risks for PPM and PVL.

Several studies have demonstrated similar mortality rates in BAV patients compared to TAVs [[22], [23], [24]]. The lower technical and device success rates observed in patients with BAV may be attributed to an increased risk of periprocedural complications. BAV patients did demonstrate numerically a higher incidence of vascular complications and major bleeding events compared to those with TAV. Furthermore, contrast usage and radiation time were higher for BAV patients, which likely reflects the higher procedural complexity commonly associated with this anatomy. Bicuspid valves often present with asymmetric and heavily calcified leaflets, elliptical annular shapes, and associated aortopathy, which can complicate both imaging interpretation and device positioning. These anatomical challenges may require repeated contrast injections for optimal visualization, more frequent repositioning of the transcatheter heart valve, and more frequent pre- and post-dilatation to achieve adequate expansion and sealing. These findings suggest a higher technical difficulty in employing TAVR in BAVs, most likely due to their different and unique anatomical characteristics. Operator experience may also play a role, as the implantation rates of TAVR in BAV remain comparatively low, leading to less familiarity and expertise with these procedures compared to TAVR in TAV patients.

In our cohort, 12 % of the BAV patients required a permanent pacemaker, and BAV anatomy was associated with an almost two-fold increase in the need for PPI compared to TAV anatomy. This finding is particularly concerning as BAV patients are typically younger, and the need for a pacemaker carries long-term implications, including an increased risk of device-related infections, repeated interventions, and potential complications such as pacing-induced cardiomyopathy. Earlier studies have found contradictory results. Makkar et al. [22] reported a PPI need of 6.2 % in BAVs with no significant difference compared to propensity-matched TAV patients. In contrast, the BIVOLUTX registry [25] reported higher incidences of PPI in BAV patients, with rates of 19 % at 30 days and 25 % at one year. These rates are higher than those generally reported for TAV patients; however, the BIVOLUTX registry did not directly compare the two groups.

The choice of transcatheter heart valve (THV) and its expansion mechanism may partly account for these observed differences. In Makkar et al.’s study, third—and fourth-generation BEV were predominantly used, whereas the BIVOLUTX registry included the SEV Evolut family of valves. Differences in valve design and their associated incidence of PPI in TAV patients are well-documented, and these may similarly apply to BAV patients. However, small observational studies have not been able to confirm this [[26], [27], [28]]. BAV morphology itself may inherently predispose patients to a higher risk of conduction disturbances compared to TAV, independent of the valve expansion mechanism. One plausible theory is the asymmetric valve expansion caused by the calcified raphe and leaflet fusion present in BAV. This structural asymmetry can lead to preferential valve expansion toward the non-coronary cusp in cases of right-left cusp fusion, exerting pressure on the atrioventricular (AV) node and thereby increasing the risk of conduction disturbances. A recent study [29] reported an increased risk of PPI in BAV patients compared to TAV patients undergoing SAVR, further supporting the notion that BAV morphology itself contributes to an elevated risk of conduction abnormalities.

Earlier studies [23,[30], [31], [32], [33]] have indicated a higher occurrence of more-than-mild PVL in BAVs, although older-generation valves were used in these trials. Several anatomical challenges unique to BAVs may increase the risk for PVL, including the heavy calcification often present, the asymmetry in leaflet size, the larger dimensions in BAVs, and the resistance to uniform device expansion during TAVR. Indeed, Yoon et al. [34] have reported that the presence of calcified raphe and excess leaflet calcification is associated with an increased risk of more-than-mild PVL and procedural complications. Recent studies employing newer-generation valves [22,24,35] have however shown a comparable short-term and long-term incidence of more-than-mild PVL while Makkar et al. [22] reported significantly lower incidences of mild PVL at 30 days with fourth-generation compared to third-generation balloon-expandable valves. Our study further provides evidence that the risk for PVL is comparable between the two valve morphologies when newer generation valves are used.

There is a growing body of evidence that TAVR is a feasible and safe option in younger BAV patients. The PARTNER 3 Bicuspid Registry [36] and Evolut Low Risk TAVR Bicuspid Study [37] included low-risk BAV patients in parallel registry-based analyses. The PARTNER 3 Bicuspid Registry reported no difference in the composite endpoint of all-cause mortality, stroke, and cardiovascular-related rehospitalization at 1 year, while the Evolut Low Risk Trial substudy demonstrated no difference in all-cause mortality or disabling stroke at 1 year. Both studies also reported comparable hemodynamic echocardiographic outcomes at 1 year. A recently published multinational observational registry [38] utilizing the Acurate neo2 valves similarly showed favourable 30 days clinical outcomes and bioprosthetic valve performance in BAV patients.

Randomized controlled trials comparing TAVR to SAVR in younger BAV patients are still sparse since BAV patients have largely been excluded from the major trials. The recent NOTION 2 trial [12], which randomized 370 patients under 75 years of age to either TAVR or SAVR, though included BAV patients (26 %). The study found that the incidence of death or disabling stroke was low in both groups, at 3.2 % for TAVR and 1.6 % for SAVR, with an absolute risk difference of 1.6 %. Sub-analyses revealed clinical equipoise between TAVR and SAVR for TAVs, with a risk difference of 0.4 percentage points for death, stroke, or re-hospitalization. However, in the bicuspid valve group, outcomes favored SAVR, with a 10.4 percentage point risk difference for the primary endpoint and a 6.1 percentage point difference for stroke. Although the study was underpowered, these findings suggest caution when considering TAVR for younger patients with BAVs.

Ultimately, the decision to treat patients with BAV stenosis using SAVR or TAVR should be highly individualized. Clinicians must consider a variety of factors beyond the immediate procedural requirements. The presence and extent of associated aortopathy and coronary artery disease that require surgical intervention can lead to a preference for SAVR. A recent study by Kedhi et al. [39], however, demonstrated favorable outcomes for percutaneous strategies over surgery in patients with severe aortic stenosis and complex coronary artery disease, including all-cause mortality, myocardial infarction, disabling stroke, clinically driven target-vessel revascularization, valve reintervention, and life-threatening or disabling bleeding at 1-year follow-up. Although promising, this study explicitly excluded patients with BAVs. As such, its findings should not be directly extrapolated to the BAV population. Further research is needed to clarify whether similar outcomes can be expected in BAV patients with complex coronary anatomy.

Furthermore, due to their typically younger age, BAV patients might outlive the longevity of bioprosthetic valves implanted via TAVR, necessitating consideration of future valve-in-valve procedures. Additionally, the need for future coronary artery access for interventions, potential requirements for pacemaker implantation and the anatomical suitability of TAVR for each patient are essential factors to evaluate in the decision-making process. Even if opting for TAVR, the choice between balloon-expandable and self-expanding valves further complicates decision-making, since each valve type may offer distinct advantages in certain patients.

Considering the increasing number of BAV stenosis patients undergoing intervention, there is a pressing need for randomized controlled trials (RCTs) to assess the safety and efficacy of TAVR compared to SAVR. These studies should particularly determine if specific BAV subpopulations might benefit more from one treatment than the other. Additionally, research should investigate the anatomical characteristics of BAV that make it more amenable to TAVR. Considering the current application of TAVR in this group, RCTs are also essential to compare the performance and outcomes of balloon-expandable versus self-expanding valves, which could provide critical insights into individualizing TAVR in each BAV patient.

## 5 Conclusions

BAV patients had comparable mortality and stroke risk to TAV patients following TAVR but demonstrated a lower rate of device success and a higher need for PPI. Despite these challenges, valve hemodynamics were similar between BAV and TAV patients, with no significant differences in the risk of PPM or PVL. These findings support TAVR as a feasible treatment option for BAV patients, offering acceptable procedural and early clinical outcomes. However, further large-scale and randomized studies are necessary to validate these results, refine patient selection, optimize procedural strategies, and assess long-term outcomes in this population.

## 6 Limitations

Despite employing robust statistical tests with a doubly robust approach and conducting sensitivity analysis, residual confounding cannot be entirely excluded. Data about each patient’s medication was missing, precluding adjustment for these variables. Furthermore, detailed information on aortic valve characteristics, such as the Sievers classification and degree of calcification, was not available in the registry. In our analysis, we defined PPM as a mean transvalvular gradient greater than 20 mmHg or Vmax over 3.5 m/s. The most widely accepted definition of PPM, as outlined by the VARC-3 criteria, is based on the indexed effective orifice area (iEOA). However, due to missing data, we were unable to calculate this parameter. Since only second-generation and later iterations of valves from the Evolut, SAPIEN, ACURATE, and Portico/Navitor families were included, the findings cannot be extrapolated to other valve manufacturers.

## CRediT authorship contribution statement

**Antros Louca:** Writing – review & editing, Writing – original draft, Software, Resources, Methodology, Funding acquisition, Formal analysis, Data curation, Conceptualization. **Petur Petursson:** Writing – review & editing, Supervision, Project administration, Methodology, Investigation, Conceptualization. **Joakim Sundström:** Writing – review & editing, Visualization. **Araz Rawshani:** Writing – review & editing, Writing – original draft, Software, Methodology, Investigation, Formal analysis, Data curation. **Henrik Hagström:** Writing – review & editing, Writing – original draft, Project administration. **Magnus Settergren:** Writing – review & editing, Conceptualization. **Stefan James:** Writing – review & editing, Supervision. **Sasha Koul:** Writing – review & editing, Visualization. **Kristofer Skoglund:** Writing – review & editing, Writing – original draft, Supervision. **Dan Ioanes:** Writing – review & editing, Writing – original draft, Investigation. **Sebastian Völz:** Writing – review & editing, Writing – original draft, Visualization, Investigation. **Anna Myredal:** Writing – review & editing, Writing – original draft, Visualization. **Oskar Angerås:** Writing – review & editing, Writing – original draft, Supervision, Conceptualization. **Truls Råmunddal:** Writing – review & editing, Writing – original draft, Supervision, Resources, Project administration, Investigation, Funding acquisition, Conceptualization.

## Funding

A.L has received funds through the Gothenburg Society of Medicine (1000894).

T.R has received funds through the Swedish Heart and Lung Foundation (20190524).

## Declaration of competing interest

The authors declare the following financial interests/personal relationships which may be considered as potential competing interests: Antros Louca reports financial support was provided by Göteborg Medical Society. Truls Ramunddal reports financial support was provided by Swedish Heart and Lung Association. Magnus Settergren reports a relationship with Abbott Vascular Inc that includes: consulting or advisory. Magnus settergren reports a relationship with Medtronic that includes: consulting or advisory. Magnus Settergren reports a relationship with Anteris Technologies Ltd that includes: consulting or advisory. Magnus settergren reports a relationship with SmartCella Holding AB that includes: consulting or advisory. Stefan James reports a relationship with Medtronic that includes: funding grants. Stefan james reports a relationship with Edwards Lifesciences Corporation that includes: funding grants. Oskar Angeras reports a relationship with Medtronic that includes: speaking and lecture fees. Oskar Angeras reports a relationship with Abbott Vascular Inc that includes: speaking and lecture fees. Oskar Angeras reports a relationship with Meril Life Sciences Private Limited that includes: speaking and lecture fees. Truls Ramunddal reports a relationship with Boston Scientific Corporation that includes: consulting or advisory. If there are other authors, they declare that they have no known competing financial interests or personal relationships that could have appeared to influence the work reported in this paper.
