# Supplementary material for: Comparative outcomes of transcatheter aortic valve replacement in bicuspid vs. tricuspid aortic valve stenosis patients: insights from the SWEDEHEART registry
Source: Int J Cardiol Heart Vasc. 2025 May 14;59:101705. doi: 10.1016/j.ijcha.2025.101705 (PMC12143612; doi:10.1016/j.ijcha.2025.101705)
Supplement: Supplementary Data 2 [file mmc2.docx]

**Supplementary Table 1. VARC-3 definitions of technical- and device success**

| **Technical success**  **(at exit from procedure room)** | Freedom f**rom mortality** |
| --- | --- |
|  | Successful access, delivery of the device, and retrieval of the delivery system |
|  | Correct positioning of a single prosthetic heart valve into the proper anatomical location |
|  | Freedom from surgery or intervention related to the device or to a major vascular or access-related, or cardiac structural complication |
| **Device success**  **(during index hospitalisation)** | Technical success |
|  | Freedom from mortality |
|  | Freedom from surgery or intervention related to the device or to a major vascular or access-related or cardiac structural complication |
|  | Intended performance of the valve (mean gradient <20 mmHg, peak velocity <3 m/s, Doppler velocity index ≥0.25, and less than moderate aortic regurgitation) |
