# Supplementary material for: Comparative outcomes of transcatheter aortic valve replacement in bicuspid vs. tricuspid aortic valve stenosis patients: insights from the SWEDEHEART registry
Source: Int J Cardiol Heart Vasc. 2025 May 14;59:101705. doi: 10.1016/j.ijcha.2025.101705 (PMC12143612; doi:10.1016/j.ijcha.2025.101705)
Supplement: Supplementary Data 3 [file mmc3.docx]

**Supplementary table 2. Missing (absolute count and percentage) in the original population**

| Outcome | Missing  (N) | Missing  (%) |
| --- | --- | --- |
| Post-TAVI average valvular gradient | 2,221 | 31.30 |
| Pacemaker following TAVI | 387 | 5.45 |
| 30-day mortality | 0 | 0 |
| All-cause mortality | 0 | 0 |
| Technical success | 0 | 0 |
| Device success | 0 | 0 |
| PPM | 0 | 0 |
| More than mild PVL | 0 | 0 |
| Stroke | 0 | 0 |
