# Supplementary material for: Comparative outcomes of transcatheter aortic valve replacement in bicuspid vs. tricuspid aortic valve stenosis patients: insights from the SWEDEHEART registry
Source: Int J Cardiol Heart Vasc. 2025 May 14;59:101705. doi: 10.1016/j.ijcha.2025.101705 (PMC12143612; doi:10.1016/j.ijcha.2025.101705)
Supplement: Supplementary Data 4 [file mmc4.docx]

**Supplementary Table 3. Balance features of the covariates in the unadjusted cohort and in the propensity score-matched cohort for tricuspid versus bicuspid aortic stenosis**

| Variable | SMD in unadjusted cohort | SMD in propensity score-matched cohort | Kolmogorov-Smirnov statistic in the unmatched cohort | Kolmogorov-Smirnov statistic in the matched cohort | Variance ratio in unmatched cohort | Variance ratio in matched cohort |
| --- | --- | --- | --- | --- | --- | --- |
| Year of TAVI: 2016 | 0.00 | 0.00 | 0.00 | 0.00 |  |  |
| Year of TAVI: 2017 | -0.03 | -0.01 | 0.03 | 0.01 |  |  |
| Year of TAVI: 2018 | -0.02 | -0.01 | 0.02 | 0.01 |  |  |
| Year of TAVI: 2019 | -0.02 | 0.00 | 0.02 | 0.00 |  |  |
| Year of TAVI: 2020 | -0.01 | 0.00 | 0.01 | 0.00 |  |  |
| Year of TAVI: 2021 | 0.02 | -0.01 | 0.02 | 0.01 |  |  |
| Year of TAVI: 2022 | 0.06 | 0.02 | **0.06** | 0.02 |  |  |
| Age at TAVI | **-0.53** | -0.03 | **0.24** | 0.03 | 1.49 | 0.95 |
| Sex: Female | -0.06 | 0.01 | **0.06** | 0.01 |  |  |
| BMI | -0.09 | 0.05 | **0.09** | 0.05 | 1.10 | 1.28 |
| NYHA functional class III or IV | -0.05 | -0.02 | **0.05** | 0.02 |  |  |
| Hypertension | -0.09 | -0.02 | **0.09** | 0.02 |  |  |
| Diabetes mellitus | -0.05 | -0.01 | **0.05** | 0.01 |  |  |
| Chronic pulmonary disease | -0.01 | -0.02 | 0.01 | 0.02 |  |  |
| Peripheral vascular disease | -0.03 | -0.01 | 0.03 | 0.01 |  |  |
| CKD | -0.08 | -0.02 | **0.08** | 0.02 |  |  |
| Atrial fibrillation | -0.08 | -0.01 | **0.08** | 0.01 |  |  |
| Myocardial infarction within 3 months | 0.00 | 0.00 | 0.00 | 0.00 |  |  |
| Previous PCI | -0.08 | 0.00 | **0.08** | 0.00 |  |  |
| Pacemaker | 0.00 | 0.00 | 0.00 | 0.00 |  |  |
| Previous cerebrovascular incident | -0.02 | 0.01 | 0.02 | 0.01 |  |  |
| Aortic valve area | 0.01 | 0.04 | 0.02 | 0.02 | 0.93 | 0.80 |
| Mean aortic valve gradient | **0.14** | 0.04 | **0.06** | **0.05** | 1.08 | 0.89 |
| Maximum aortic valve gradient | **0.15** | 0.03 | **0.07** | 0.04 | 1.16 | 0.96 |
| LVEF: HFmrEF | -0.01 | -0.02 | 0.01 | 0.02 |  |  |
| LVEF: HFrEF | 0.05 | -0.01 | **0.05** | 0.01 |  |  |
| LVEF: Normal EF | -0.03 | 0.04 | 0.03 | 0.04 |  |  |
| Moderate/severe aortic insufficiency | 0.01 | 0.00 | 0.01 | 0.00 |  |  |
| Moderate/severe mitral insufficiency | -0.03 | -0.02 | 0.03 | 0.02 |  |  |
| Annular diameter | **0.51** | -0.02 | **0.23** | 0.02 | 1.21 | 1.02 |
| Pulmonary hypertension | **-0.21** | 0.02 | **0.10** | 0.03 | 1.00 | 1.03 |
| Porcelain aorta | -0.02 | 0.01 | 0.02 | 0.01 |  |  |
| Thorax deformity | 0.01 | 0.01 | 0.01 | 0.01 |  |  |
| Unfavorable anatomy | -0.01 | 0.01 | 0.01 | 0.01 |  |  |
| TAVI urgency: Urgent | 0.03 | 0.01 | 0.03 | 0.01 |  |  |
| Access site: Direct aortic access | 0.00 | 0.00 | 0.00 | 0.00 |  |  |
| Access site: Transapical | -0.01 | 0.00 | 0.01 | 0.00 |  |  |
| Access site: Transfemoral | 0.01 | 0.01 | 0.01 | 0.01 |  |  |
| Access site: Via Subclavian artery | 0.00 | -0.01 | 0.00 | 0.01 |  |  |
| BEV/SEV: SEV | -0.03 | 0.01 | 0.03 | 0.01 |  |  |

Significant differences are highlighted in bold using the following thresholds: a standardized mean difference of 0.10, a Kolmogorov-Smirnov statistic of 0.05, and variance ratios of 2.
