# Supplementary material for: Comparative outcomes of transcatheter aortic valve replacement in bicuspid vs. tricuspid aortic valve stenosis patients: insights from the SWEDEHEART registry
Source: Int J Cardiol Heart Vasc. 2025 May 14;59:101705. doi: 10.1016/j.ijcha.2025.101705 (PMC12143612; doi:10.1016/j.ijcha.2025.101705)
Supplement: Supplementary Data 5 [file mmc5.docx]

**Supplementary Table 4:** Comparison of Selected Outcomes for Technical Success Failure Between Bicuspid and Tricuspid Aortic Valve Stenosis.

| **Outcome** | **Bicuspid**  **aortic valve stenosis**  **N = 577** | **Tricuspid**  **aortic valve stenosis**  **N = 6,518** |
| --- | --- | --- |
| Deceased in procedure room | 2 (0.3%) | 20 (0.3%) |
| Procedure failure  (Operator assessment) | 26 (4.5%) | 181 (2.8%) |
| Cardiac tamponade | 2 (0.3%) | 42 (0.6%) |
| Major bleeding | 20 (3.5%) | 201 (3.1%) |
| Vascular complication |  |  |
| Aorta | 4 (0.7%) | 14 (0.2%) |
| Access site | 8 (1.4%) | 93 (1.4%) |
| Other | 1 (0.2%) | 8 (0.1%) |
| Annulus rupture | 0 (0%) | 7 (0.1%) |
| Conversion to heart operation | 1 (0.2%) | 11 (0.2%) |
| Need for vascular intervention | 6 (1.0%) | 55 (0.8%) |
| Coronary occlusion | 1 (0.2%) | 8 (0.1%) |
| Additional valve needed | 10 (1.7%) | 68 (1.0%) |
| Valve embolization |  |  |
| To aorta | 5 (0.9%) | 31 (0.5%) |
| To left ventricle | 0 (0%) | 6 (<0.1%) |
|  | | |
