# Supplementary material for: Comparative outcomes of transcatheter aortic valve replacement in bicuspid vs. tricuspid aortic valve stenosis patients: insights from the SWEDEHEART registry
Source: Int J Cardiol Heart Vasc. 2025 May 14;59:101705. doi: 10.1016/j.ijcha.2025.101705 (PMC12143612; doi:10.1016/j.ijcha.2025.101705)
Supplement: Supplementary Data 6 [file mmc6.docx]

Supplementary Table 5. Comparison of Selected Outcomes for Device Failure during Index Hospitalization between Bicuspid and Tricuspid Aortic Valve Stenosis.

| **Outcome** | **Bicuspid**  **aortic valve stenosis**  **N = 577** | **Tricuspid**  **aortic valve stenosis**  **N = 6,518** |
| --- | --- | --- |
| Technical failure | 66 (11%) | 522 (8.0%) |
| Death during index hospitalization | 4 (0.7%) | 67 (1.0%) |
| Cardiac tamponade | 6 (1.0%) | 36 (0.6%) |
| Major bleeding | 20 (3.5%) | 201 (3.1%) |
| Vascular complication | 6 (1.0%) | 107 (1.6%) |
| More-than-mild PVL | 25 (4.3%) | 162 (2.5%) |
| Prosthesis-patient mismatch | 19 (3.3%) | 207 (3.2%) |
| PVL: paravalvular leakage  **Note:** The absolute counts and percentages for each complication are calculated from the time the patient exits the procedure room until the discharge date. Complications that occur within the procedure room are detailed in Supplementary Table 4. | | |
